# Prenatal Exposure to Organophosphate Pesticides and IQ in 7-Year-Old Children

**DOI:** 10.1289/ehp.1003185

**Published:** 2011-04-21

**Authors:** Maryse F. Bouchard, Jonathan Chevrier, Kim G. Harley, Katherine Kogut, Michelle Vedar, Norma Calderon, Celina Trujillo, Caroline Johnson, Asa Bradman, Dana Boyd Barr, Brenda Eskenazi

**Affiliations:** 1 University of California–Berkeley, Center for Environmental Research and Children’s Health, School of Public Health, Berkeley, California, USA; 2 CHU Sainte-Justine Research Center and Université de Montréal, Department of Environmental and Occupational Health, Montreal, Quebec, Canada; 3 Center for the Health Assessment of Mothers and Children of Salinas (CHAMACOS), Clinica de Salud del Valle de Salinas, Salinas, California, USA; 4 Emory University, Rollins School of Public Health, Atlanta, Georgia, USA

**Keywords:** agriculture, children, cognitive development, farmworker, insecticides, intelligence quotient, neurodevelopment, organophosphate, pesticides

## Abstract

Context: Organophosphate (OP) pesticides are neurotoxic at high doses. Few studies have examined whether chronic exposure at lower levels could adversely affect children’s cognitive development.

Objective: We examined associations between prenatal and postnatal exposure to OP pesticides and cognitive abilities in school-age children.

Methods: We conducted a birth cohort study (Center for the Health Assessment of Mothers and Children of Salinas study) among predominantly Latino farmworker families from an agricultural community in California. We assessed exposure to OP pesticides by measuring dialkyl phosphate (DAP) metabolites in urine collected during pregnancy and from children at 6 months and 1, 2, 3.5, and 5 years of age. We administered the Wechsler Intelligence Scale for Children, 4th edition, to 329 children 7 years of age. Analyses were adjusted for maternal education and intelligence, Home Observation for Measurement of the Environment score, and language of cognitive assessment.

Results: Urinary DAP concentrations measured during the first and second half of pregnancy had similar relations to cognitive scores, so we used the average of concentrations measured during pregnancy in further analyses. Averaged maternal DAP concentrations were associated with poorer scores for Working Memory, Processing Speed, Verbal Comprehension, Perceptual Reasoning, and Full-Scale intelligence quotient (IQ). Children in the highest quintile of maternal DAP concentrations had an average deficit of 7.0 IQ points compared with those in the lowest quintile. However, children’s urinary DAP concentrations were not consistently associated with cognitive scores.

Conclusions: Prenatal but not postnatal urinary DAP concentrations were associated with poorer intellectual development in 7-year-old children. Maternal urinary DAP concentrations in the present study were higher but nonetheless within the range of levels measured in the general U.S. population.

Organophosphate (OP) pesticides are widely used in agriculture, and several are registered for home garden use [U.S. Environmental Protection Agency (EPA) 2006]. In 2010, 32 OP pesticides were registered in the United States (U.S. EPA 2010). In 2007, 15 million kilograms of OP pesticides were used in the United States, representing 36% of all insecticides used (Grube et al. 2011). In California, 1.6 million kilograms of OP pesticides were used in agriculture in 2008; the top five active ingredients were chlorpyrifos, malathion, phosmet, ethephon, and dimethoate [California Department of Pesticide Regulation (CDPR) 2010].

OP pesticides have well-known neurotoxic properties, with the primary mechanism of action involving inhibition of acetylcholinesterase at high doses (Sultatos 1994). At doses lower than those needed to inhibit acetylcholinesterase, certain OP pesticides affect different neurochemical targets, including growth factors, several neurotransmitter systems, and second-messenger systems (Slotkin and Seidler 2007; Verma et al. 2009).

Most human studies showing adverse health effects of OP pesticides have been carried out in occupational settings with high exposure levels (Kamel et al. 2007). Children may experience chronic, low-level exposure due to historical home use, living near an agricultural field, and residues in food (Bradman et al. 2007; Lu et al. 2004). Children are at higher risk for pesticide toxicity than are adults because the developing brain is more susceptible to neurotoxicants and the dose of pesticides per body weight is likely to be higher in children (Weiss 2000). Children also have lower activity and levels of enzymes that detoxify activated forms of certain OP pesticides (Holland et al. 2006).

Epidemiologic studies suggest that prenatal exposure to OP pesticides is associated with poorer neurobehavioral development in infants (Engel et al. 2007; Young et al. 2005) and toddlers and preschoolers (Eskenazi et al. 2007; Handal et al. 2008; Rauh et al. 2006). Postnatal OP exposure has also been associated with behavioral problems; poorer short-term memory, executive function, and motor skills; and longer reaction time in children (Bouchard et al. 2010; Grandjean et al. 2006; Rohlman et al. 2005; Ruckart et al. 2004). Few studies have assessed exposure to OP pesticides both prenatally and during childhood.

The Center for the Health Assessment of Mothers and Children of Salinas (CHAMACOS) study is a birth cohort study investigating pesticide and other environmental exposures and the health of pregnant women and their children living in an agricultural community. Our findings suggest that most maternal pesticide exposure probably occurs through the diet, as is the case for the general U.S. population, but with additional residential nondietary exposure most likely from ingress of pesticides from agricultural use into homes (Harnly et al. 2009; McKone et al. 2007). Previous reports on the CHAMACOS cohort suggested that prenatal, but not postnatal, exposure to OP pesticides was associated with increased odds of pervasive developmental disorder and lower scores of mental development at 2 years of age (Eskenazi et al. 2007), and with poorer attention skills as well as hyperactive behaviors at 5 years of age (Marks et al. 2010). It remains unclear whether cognitive deficits associated with prenatal exposure to OP pesticides are persistent, because cohort studies have not followed children to school age, when deficits may have greater implications for school performance. Here, we report the association between prenatal and postnatal exposure to OP pesticides, indicated by urinary dialkyl phosphate (DAP) metabolite concentrations, and cognitive abilities of 7-year-olds.

## Participants and Methods

*Study setting and design.* The CHAMACOS study is a community/university partnership. We conducted this longitudinal birth cohort study of predominantly Mexican-American low-income families living in the Salinas Valley, California. This agricultural region is located southeast of the San Francisco Bay Area. Common crops include lettuce, strawberries, artichokes, broccoli, and grapes. About 235,000 kg of OP pesticides were used in this region in 1999–2000, when study participants were pregnant (CDPR 2001). Current data show that use of OP pesticides in California is decreasing overall; however, use of OP pesticides in Monterey County remained steady between 2001 and 2008 but declined 18% from 2008 to 2009 (CDPR 2010).

Detailed methods for the CHAMACOS study have been described elsewhere (Eskenazi et al. 2004, 2006). Briefly, pregnant women were recruited in six community clinics, serving primarily farmworker families, between October 1999 and 2000. Eligible women were ≥ 18 years old, < 20 weeks of gestation, Spanish or English speaking, eligible for low-income health insurance, and planning to deliver at the local public hospital. All study activities were approved by the University of California–Berkeley Committee for the Protection of Human Subjects. Written, informed consent was obtained from the mothers, and child assent was obtained at 7 years of age.

The initial cohort included 601 women who delivered 526 live-born surviving singletons. For the present study, we excluded two children with missing prenatal DAP concentration measurements, four children with a medical condition that would affect assessment (autism, Down syndrome, hydrocephalus, deafness), children who were lost to follow-up and/or did not participate at the 7-year study visit (*n* = 72 moved, *n* = 59 refused, *n* = 24 unable to trace, *n* = 21 unable to schedule, *n* = 2 deceased), and children missing the cognitive assessment at the 7-year visit (*n* = 13). Families included in this analysis (*n* = 329) did not differ significantly from the original full cohort on most attributes, including urinary DAP concentrations during pregnancy, maternal measures of cognitive ability, maternal education, marital status, poverty category, and child’s birth weight. However, mothers of children included in the present study were slightly older (mean age, 26.7 vs. 26.0 years, *p* = 0.07) and breast-fed longer (8.7 months vs. 7.2, *p* = 0.01) than those from the initial cohort.

*Cognitive assessment.* We used the Wechsler Intelligence Scale for Children, 4th edition (WISC-IV), to assess cognitive abilities at the 7-year study visit (Wechsler 2003). All assessments were completed by a single experienced bilingual psychometrician, who was trained and supervised by a pediatric neuropsychologist. Quality assurance measures included review of videotaped assessments. For subtests in which a ceiling was not achieved (< 10%), missing values were imputed based on scores obtained by other children with similar score patterns. Scores for four domains were calculated based on the following subtests: Verbal Comprehension (composed of the Vocabulary and Similarities subtests), Perceptual Reasoning (Block Design and Matrix Reasoning subtests), Working Memory (Digit Span and Letter-Number Sequencing subtests), and Processing Speed (Coding and Symbol Search subtests). All subtests were administered in the dominant language of the child, which was determined through administration of the oral vocabulary subtest of the Woodcock–Johnson/Woodcock–Muñoz Tests of Cognitive Ability in both English and Spanish (Woodcock and Johnson 1990). Ultimately, 67% of children were tested in Spanish and 33% in English. WISC-IV scores are standardized against U.S. population–based norms for English- and Spanish-speaking children.

The numbers of children with available scores were 329 for Perceptual Reasoning and Verbal Comprehension and 298 for Processing Speed and Working Memory (because we did not administer letter-number sequencing and symbol search for the first 3 months of assessments). A Full-Scale intelligence quotient (IQ) was available for 297 children.

*Maternal interviews and assessments.* Women were interviewed twice during pregnancy (median gestation, 13 and 26 weeks), after delivery, and when children were 6 months and 1, 2, 3.5, 5, and 7 years of age. Interviews were conducted in Spanish or English by bilingual, bicultural interviewers. At the 6-month visit, mothers were administered the Peabody Picture Vocabulary Test (PPVT) to assess verbal intelligence (Dunn and Dunn 1981). The Infant-Toddler HOME (Home Observation for Measurement of the Environment) inventory was completed at 6 months and 1 and 2 years of age, and a short version was completed at 3.5 and 5 years (Caldwell and Bradley 1984). Additional information was obtained from prenatal and delivery medical records, which was abstracted by a registered nurse.

*Urinary OP metabolite measurements.* Urine was collected at two time points during pregnancy. The first urine sample was collected at enrollment into the study, between 5 and 27 weeks of gestation (median, 13 weeks). The second urine sample was collected between 18 and 39 weeks (median, 26 weeks). Urine was collected from the children at 6 months and 1, 2, 3.5, and 5 years of age; no urine was collected at the 7-year visit.

Urine specimens were aliquoted and stored at −80°C until shipment on dry ice to the Centers for Disease Control and Prevention (CDC; Atlanta, GA) for analysis. Six nonspecific OP DAP metabolites were measured in maternal and child urine: three dimethyl (DM) phosphate metabolites (dimethylphosphate, dimethylthiophosphate, dimethyldithiophosphate) and three diethyl (DE) phosphate metabolites (diethylphosphate, diethylthiophosphate, and diethyldithiophosphate). These six metabolites cannot be traced back to individual pesticides but together represent the breakdown products of about 80% of the total OP pesticides used in the Salinas Valley (CDC 2009). The most commonly used OP pesticides in the Salinas Valley are chlorpyrifos and diazinon (which devolve to DE), as well as malathion and oxydemeton-methyl (which devolve to DM). DAP metabolite concentrations were measured using gas chromatography/tandem mass spectrometry and quantified using isotope dilution calibration (Bravo et al. 2002). Details of urine collection, analysis, detection frequencies, and quality control procedures are described elsewhere (Bradman et al. 2005). Concentrations below the limit of detection (LOD) were randomly imputed based on a log-normal probability distribution whose parameters were estimated using maximum likelihood estimation. This method has been shown to perform better than simple substitution methods such as LOD/2 or LOD/_√_^–^2 (Lubin et al. 2004). The DAP metabolite concentrations were expressed on a molar basis and summed to yield total DE, DM, and DAP concentrations.

*Other environmental contaminants.* We also considered the potential confounding effects of other known or suspected neurotoxicants: polybrominated diphenyl ethers (PBDEs), polychlorinated biphenyls (PCBs), *p*,*p*´-dichlorodiphenyltrichloroethane (DDT), *p*,*p*´-dichlorodiphenyldichloroethylene (DDE), and lead. Lead was measured in maternal blood at 26 weeks of gestation, in cord blood for a subset of the participants by the California Department of Public Health (Richmond, CA, USA), and in children’s blood at 2 years of age by the Monterey County Public Health Laboratory (Salinas, CA, USA), using graphite furnace atomic absorption spectrophotometry. PBDEs, PCBs, DDT, and DDE were measured by the CDC (Atlanta, GA) in maternal serum samples collected at 26 weeks of gestation, on average, using gas chromatography/isotope-dilution high-resolution mass spectrometry and were expressed on a serum lipid basis. Total lipids were determined based on the measurement of triglyceride and total cholesterol in serum using standard enzymatic methods (Roche Chemicals, Indianapolis, IN) (Phillips et al. 1989).

*Data analysis.* Nonspecific total DAP, DE, and DM metabolites (nanomoles per liter) were transformed to the log_10_ scale. All analyses were conducted on non-creatinine-adjusted values; models were rerun with creatinine-adjusted values (nanomoles per gram of creatinine) in sensitivity analyses. We examined the association between urinary DAP concentrations and cognitive scores using multiple linear regression, with point estimates representing the change in cognitive scores for each 10-fold increase in DAP concentrations. For prenatal exposure, we examined associations with the DAP concentrations measured separately for urine collected during the first and second half of pregnancy (≤ 20 vs. > 20 weeks of gestation). Because we found similar relations between cognitive scores and DAPs measured earlier or later than 20 weeks of gestation, we averaged the two DAP measures for further analyses; for 20 children (6%) only one prenatal measure was available for the analyses. Because DM and DE metabolites might have different relationships to the outcomes, they were examined separately.

For postnatal exposure, we examined the cross-sectional association of cognitive scores with DAP concentrations measured in children’s urine collected at different ages in separate models. We also calculated the cumulative DAP level between 6 and 60 months using the area under the curve (AUC), calculated using the trapezoidal method. For 46 children with one missing DAP measurement at 1, 2, or 3.5 years, we imputed the mean of the two measures closest in time for the AUC calculation. Forty-nine children who were missing DAP measures at either the 6-month or 5-year visit, or missing more than one DAP measure from the three other time points, were excluded from the AUC analysis. For comparison with prenatal exposure, we calculated the mean urinary DAP concentrations measured during childhood for children with at least three of five measures (taken at 6 months and 1, 2, 3.5, and 5 years); this excluded 20 children.

To explore possible synergistic effects between pre- and postnatal DAP concentrations, we included an interaction term for mean prenatal DAP concentrations × AUC. However, this term was not statistically significant (*p* > 0.15) and thus was not included in the final models.

We retained the following variables as covariates for all analyses: maternal intellectual abilities (PPVT score, continuous), maternal education (three categories), and continuous HOME score at 6 months. Maternal intellectual abilities and HOME score were included in models because they were associated with both DAP concentrations and IQ scores in univariate analyses (*p* < 0.2), and maternal education was included because it is an important determinant of children’s cognitive development. Language of testing was also included in models for Verbal Comprehension and Full-Scale IQ because of observed language-related differences in scores for these scales. We conducted additional analyses to evaluate the confounding effect of other factors associated with neurodevelopment in the literature: breast-feeding duration (in weeks, continuous), maternal age (continuous), birth order (continuous), HOME score at 1, 2, 3.5, and 5 years (continuous), poverty category (coded as in Table 1), marital status (married or living as married/not married), children’s age at WISC-IV testing (in months, continuous), and maternal levels of PBDEs, PCBs, DDE, DDT, and lead during pregnancy. Each of these variables was added individually to the final model, but none was retained because none changed the magnitude of the coefficient for urinary DAP concentrations by > 10%. In separate analyses, we also investigated potential confounding and effect modification by variables possibly on the causal pathway (i.e., birth weight and gestational age, assessed continuously). Because most children (67%) were tested in Spanish, we reran the analyses restricted to this subset. Finally, we examined the interaction between sex and DAP concentrations, based on previous findings in this cohort (Marks et al. 2010).

**Table 1:** Study cohort characteristics and maternal urinary DAP concentrations (mean of two measures taken during pregnancy), CHAMACOS (*n* = 329).

Cohort characteristic	*n* (%)	Geometric mean DAP (95% CI) (nmol/L)	*p*-Value*a*
Child’s sex						0.50
Boys		154 (47)		136 (115–161)		
Girls		175 (53)		126 (108–146)		
Maternal education						0.71
< 6th grade		148 (45)		127 (106–152)		
7th–12th grade		111 (34)		127 (105–154)		
Completed high school		70 (21)		143 (113–181)		
Maternal intelligence (PPVT score)						0.17
≤ 74		106 (32)		150 (121–188)		
75–99		120 (36)		129 (109–154)		
≥ 100		103 (31)		103 (95–136)		
HOME score at 6 months						0.10
≤ 31.0		139 (42)		150 (128–177)		
31.1–33.3		85 (26)		118 (95–147)		
≥ 33.4		105 (32)		117 (97–141)		
Family income at 7 years						0.31
< Poverty level		232 (71)		125 (109–142)		
Within 200% of poverty level		95 (29)		143 (116–179)		
> 200% of poverty level		2 (1)		283 (207–389)		
Language of WISC-IV verbal subtests						0.77
English		108 (33)		133 (108–160)		
Spanish		221 (67)		129 (113–149)		
Maternal country of birth						0.55
Mexico		282 (86)		133 (118–152)		
United States		43 (13)		115 (83–160)		
Other		4 (1)		94 (62–186)		
Mother performed farm work during pregnancy						0.64
Yes		145 (44)		123 (105–146)		
No		180 (55)		136 (117–159)		
Missing		4 (1)		160 (31–2539)		
Farmworker in household during pregnancy						0.76
Yes		273 (83)		128 (113–145)		
No		54 (16)		142 (110–189)		
Missing		2 (1)		108 (89–131)		
**a**One-way analysis of variance on log_10_-transformed DAP concentrations.						

We compared effect estimates for urinary DAPs measured in early versus late pregnancy and in the prenatal versus postnatal periods using seemingly unrelated estimation (Weesie 1999); we used the mean postnatal DAP concentrations (as opposed to AUC) for these analyses in order to compare metrics with similar units. We used generalized additive models with 3-degree-of-freedom cubic splines to evaluate the shape of dose–response curves, test the linearity assumption, and investigate potential thresholds while controlling for covariates. We did not observe evidence of departure from linearity or threshold for effect, so we retained the simpler models based on linear regression. For illustration, we grouped DAP concentrations into quintiles, entered this categorical variable in the multiple regression model with the same covariables described above, and obtained the mean IQ score for each quintile.

Univariate and multiple linear regression analyses were conducted with SPSS (version 19.0; IBM Corp., Somers, NY), and generalized additive model and seemingly unrelated estimation (“suest” command) were performed with STATA (version 10.1; StataCorp, College Station, TX).

## Results

Most women in the present study were Spanish speaking, were born in Mexico, lived in farmworker households, did not complete high school, and had a family income below the U.S. poverty threshold (Table 1); 44% of mothers performed agricultural work during their pregnancy. Additional descriptive characteristics on this study population can be found elsewhere (Eskenazi et al. 2004). Levels of urinary DAP metabolites during pregnancy (mean of the two prenatal measures) were not associated with maternal education or intelligence, family income, or the language of children’s cognitive assessment (Table 1). However, DAP levels during pregnancy were higher among mothers from families with lower HOME scores (indicative of a less favorable home environment) at the child’s 6-month visit (*p* = 0.10).

DAP concentrations in urine collected during the first half (median, 13th week of gestation) and second half (median, 26th week of gestation) of pregnancy were associated with lower cognitive scores on all subtests in children at 7 years of age but were most strongly associated for Verbal Comprehension and Full-Scale IQ (Table 2). The effect coefficients for DAP concentrations measured in the first half of pregnancy were not significantly different from those for the second half of pregnancy (similarly unrelated regression, *p* > 0.05 for all scales).

**Table 2:** Change in cognitive scores in children tested at 7 years of age, for a 10-fold increase in maternal DAP concentrations (nmol/L) measured in the first and second half of pregnancy (≤ 20 weeks, > 20 weeks), CHAMACOS.

First half of pregnancy					Second half of pregnancy				
Cognitive test	*n*	β-Coefficient (95% CI)	*p*-Value	*n*	β-Coefficient (95% CI)	*p*-Value
WISC-IV scale												
Working Memory		267		–1.6 (–4.2 to 1.0)		0.22		279		–3.0 (–6.4 to 0.4)		0.08
Processing Speed		268		–1.5 (–3.9 to 0.9)		0.21		280		–2.6 (–5.9 to 0.7)		0.12
Verbal Comprehension		291		–2.6 (–5.1 to –0.1)		0.04		309		–3.1 (–6.4 to 0.2)		0.06
Perceptual Reasoning		292		–1.2 (–4.1 to 1.7)		0.42		309		–2.4 (–6.3 to 1.4)		0.22
Full-scale IQ		266		–2.4 (–4.9 to 0.2)		0.07		279		–3.5 (–6.9 to –0.1)		0.04
Estimates were adjusted for HOME score at 6 months and maternal education and intelligence. Verbal Comprehension and Full-Scale IQ were also adjusted for language of assessment.												

**Figure 1:**
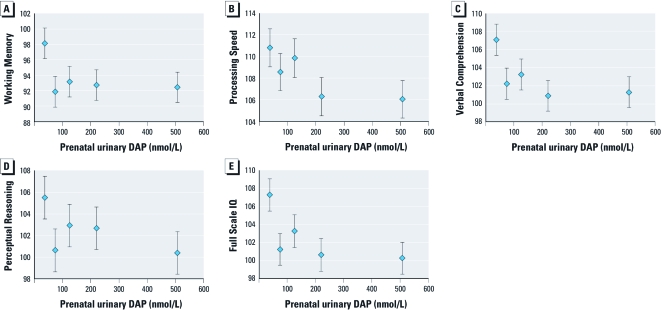
Mean ± SE WISC-IV score per quintile of prenatal urinary DAP concentration: Working Memory (*A*), Processing Speed (*B*), Verbal Comprehension (*C*), Perceptual Reasoning (*D*), and Full-Scale IQ (*E*). The medians (ranges) for DAP quintiles (nmol/L) are as follows: first quintile, 39 (0–55); second, 75 (> 55–93); third, 126 (> 93–173); fourth, 221 (> 173–319); and fifth, 508 (> 319). Estimates were adjusted for HOME score at 6 months, maternal education and intelligence, and language of assessment (only for Verbal Comprehension and Full Scale IQ).

Averaging the two maternal urinary DAP concentrations measured during pregnancy yielded significant associations with poorer cognitive scores (Table 3). Higher prenatal DAP concentrations were associated with lower scores on all four cognitive domains, the strongest associations being for Verbal Comprehension [β for a 10-fold increase in concentration = –5.3; 95% confidence interval (CI), –8.6 to –2.0]. A 10-fold increase in DAP concentrations was associated with a decrease of 5.6 Full-Scale IQ points (95% CI, –9.0 to –2.2). We did not observe evidence of departure from linearity in the relation between DAP concentrations and Full-Scale IQ. We found a 7.0 Full-Scale IQ–point difference between children in the highest quintile of prenatal DAP levels and those in the lowest quintile (Figure 1). Urinary DM concentrations averaged during pregnancy were also associated with poorer cognitive scores, although point estimates were slightly smaller than for total DAP concentrations (Table 3). Urinary DE concentrations were associated with poorer cognitive score but much less strongly than were total DAP and DM concentrations. The exception was Processing Speed scores, which were more strongly associated with DE concentrations (β: –4.0; 95% CI, –7.0 to –1.0) than with DM concentrations.

**Table 3:** Change in cognitive scores in children tested at 7 years of age, for a 10-fold increase in maternal DAP, DM, and DE concentrations (nmol/L) averaged over pregnancy, CHAMACOS.

DAP			DM			DE		
Cognitive test	*n*	β-Coefficient (95% CI)	*p*-Value	β-Coefficient (95% CI)	*p*-Value	β-Coefficient (95% CI)	*p*-Value
WISC-IV scale														
Working Memory		298		–4.3 (–7.7 to –0.9)		0.01		–4.0 (–7.1 to –1.0)		< 0.01		–0.4 (–3.5 to 2.7)		0.80
Processing Speed		298		–3.4 (–6.8 to –0.1)		0.04		–1.8 (–4.8 to 1.2)		0.23		–4.0 (–7.0 to –1.0)		< 0.01
Verbal Comprehension		329		–5.3 (–8.6 to –2.0)		< 0.01		–4.8 (–7.8 to –1.9)		< 0.01		–2.0 (–5.0 to 1.1)		0.20
Perceptual Reasoning		329		–4.0 (–7.9 to –0.1)		0.04		–3.3 (–6.7 to 0.2)		0.07		–2.1 (–5.6 to 1.5)		0.25
Full-scale IQ		297		–5.6 (–9.0 to –2.2)		< 0.01		–4.7 (–7.7 to –1.6)		< 0.01		–2.8 (–5.6 to 0.3)		0.08
Estimates were adjusted for HOME score at 6 months and maternal education and intelligence. Verbal Comprehension and Full-Scale IQ were also adjusted for language of assessment.														

We examined the confounding and effect modification of potential neurotoxicants (i.e., PBDEs, PCBs, DDT/DDE, and lead) on Full-Scale IQ, and none confounded or interacted with the DAP prenatal measurements. Adjusting for postnatal urinary DAP measures using AUC altered the results for prenatal DAPs only marginally (change in estimates < 10%). We found no indication of sex-related differential associations of cognitive scores and prenatal DAP, DM, and DE concentrations (*p* for interaction term > 0.3). Adjusting point estimates for sex only marginally altered the results. Point estimates for creatinine-adjusted DAP concentrations [Supplemental Material, Table 1 (doi:10.1289/ehp.1003185)] were similar to those obtained using non-creatinine-adjusted concentrations. Restricting the analyses to children tested in Spanish yielded results similar to those obtained for the entire group [Supplemental Material, Table 2 (doi:10.1289/ehp.1003185)].

Postnatal urinary DAP concentrations were not consistently associated with cognitive scores at 7 years of age (Table 4). However, DAP concentrations at 1 year of age were associated with better scores on Verbal Comprehension (β = 2.9; 95% CI, 0.7–5.2) and Full-Scale IQ (β =: 2.7; 95% CI, 0.3–5.1). DAP concentrations at 6 months and 2, 3.5, and 5 years, and cumulated throughout childhood by the AUC method, were not associated with cognitive scores. Likewise, postnatal DM and DE concentrations were not associated with cognitive scores (data not shown). Adjusting for prenatal urinary DAP measure had a negligible effect on the postnatal DAP results (change in estimates < 10%).

**Table 4:** Change in cognitive scores for a 10-fold increase in DAP concentrations (nmol/L) measured throughout children’s follow-up visits and for the AUC, CHAMACOS.

6 months			12 months			24 months			42 months			60 months			AUC		
Cognitive test (WISC-IV scale)	*n*	β-Coefficient (95% CI)	*p*-Value	*n*	β-Coefficient (95% CI)	*p*-Value	*n*	β-Coefficient (95% CI)	*p*-Value	*n*	β-Coefficient (95% CI)	*p*-Value	*n*	β-Coefficient (95% CI)	*p*-Value	*n*	β-Coefficient (95% CI)	*p*-Value
Working Memory		265	–1.7 (–3.9 to 0.5)	0.13		274	0.9 (–1.4 to 3.2)	0.46		274	–0.4 (–2.7 to 1.9)	0.73		231	0.8 (–1.7 to 3.3)	0.51		273	2.0 (–0.1 to 4.0)	0.07		245	1.6 (–2.2 to 5.4)	0.40
Processing Speed		266	–0.3 (–2.5 to 1.8)	0.77		274	1.6 (–0.6 to 3.8)	0.16		274	–2.0 (–4.3 to 0.2)	0.08		231	–1.1 (–3.6 to 1.3)	0.36		273	0.7 (–1.3 to 2.7)	0.48		246	–1.3 (–4.9 to 2.3)	0.49
Verbal Comprehension		294	0.8 (–1.4 to 3.0)	0.47		303	2.9 (0.7 to 5.2)	0.01		303	–0.8 (–3.1 to 1.5)	0.50		259	0.2 (–2.2 to 2.6)	0.86		302	0.4 (–1.6 to 2.5)	0.68		271	0.8 (–3.0 to 4.6)	0.67
Perceptual Reasoning		294	–2.4 (–4.9 to 0.1)	0.06		303	1.9 (–0.8 to 4.5)	0.16		303	–0.7 (–3.4 to 2.0)	0.61		259	–0.3 (–3.0 to 2.5)	0.85		302	2.3 (–0.1 to 4.7)	0.06		271	0.5 (–3.8 to 4.8)	0.81
Full-Scale IQ		265	–0.9 (–3.2 to 1.3)	0.41		273	2.7 (0.3 to 5.1)	0.03		273	–1.5 (–3.9 to 0.9)	0.22		231	0.2 (–2.4 to 2.8)	0.90		272	1.7 (–0.4 to 3.9)	0.12		245	0.6 (–3.2 to 4.4)	0.75
Estimates were adjusted for HOME score at 6 months and maternal education and intelligence. Verbal Comprehension and Full-Scale IQ were also adjusted for language of assessment.																								

To compare the association between cognitive scores and DAP concentrations measured pre- and postnatally, we restricted the sample to children with measures available for both the prenatal and postnatal periods [Supplemental Material, Table 3 (doi:10.1289/ehp.1003185)]. The effect coefficients associated with prenatal DAP concentrations (mean of two measures) were significantly different than those associated with DAP concentrations measured postnatally (mean of five measures) for Verbal Comprehension (*p* = 0.01) and Full-Scale IQ (*p* = 0.03). We observed similar findings for the other cognitive scales, but the difference between prenatal and postnatal point estimates did not reach significance (*p* > 0.10).

## Discussion

Our findings suggest that prenatal exposure to OP pesticides, as measured by urinary DAP metabolites in women during pregnancy, is associated with poorer cognitive abilities in children at 7 years of age. Children in the highest quintile of maternal DAP concentrations had an average deficit of 7.0 IQ points compared with those in the lowest quintile. Associations were linear, and we observed no threshold. However, DAP concentrations during childhood were not associated with cognitive scores in this cohort of children.

Developing fetal nervous systems may be more vulnerable to *in utero* exposure to OP pesticides because of the many unique processes occurring during this stage of development, such as cell division, migration, differentiation, formation of synapses, pruning of synapses, apoptosis, and myelination (Tau and Peterson 2010). Fetal exposure to OP pesticides occurs via passage of the OPs through the placenta (Rauh et al. 2006; Whyatt et al. 2009). In addition, DAP metabolites have been detected in amniotic fluid (Bradman et al. 2003).

Previous reports from this cohort have also shown associations of prenatal but not postnatal OP exposure with adverse neurobehavioral functioning (Eskenazi et al. 2007; Marks et al. 2010). Our findings are consistent with those of other investigations of adverse associations between prenatal exposure to OP pesticides and cognition (Harari et al. 2010; Rauh et al. 2006). In contrast to the present findings, a few other studies reported that OP metabolites measured in children were associated with poorer cognitive abilities (Lizardi et al. 2008; Ruckart et al. 2004). However, these studies differed in exposure and/or the outcomes found to be associated with OP pesticides. For instance, Ruckart et al. (2004) examined the relationship between methyl parathion—an OP pesticide rarely used in the Salinas Valley—and found no association with general intelligence in 6-year-olds but did find adverse associations between concurrent exposure and other specific neuropsychological domains (i.e., poorer memory, attention, and motor skills). In a study of 48 children 7 years of age, Lizardi et al. (2008) reported that those with detectable levels of DAPs had a worse performance on a test of executive function, but not on the Full-Scale IQ, compared with those with nondetectable levels. Studies of women who worked in floriculture in Ecuador found associations with certain specific neurobehavioral domains in their children but did not assess general intelligence (Grandjean et al. 2006; Handal et al. 2007, 2008; Harari et al. 2010).

This study has limitations, mostly related to the assessment of exposure to OP pesticides. Assessing OP exposure is challenging because of their fast clearance from the body, with complete excretion in the urine within 3–6 days (Bradway et al. 1977). We observed that prenatal exposure indicated by the average of two DAP metabolite measures taken during pregnancy was associated with markedly poorer cognitive performances. However, the association of DAP metabolites measured at one point in time during pregnancy—either earlier or later during gestation—was not as strongly associated with cognitive scores. Considering the rapid metabolism of these compounds, it seems likely that exposure assessment based on a single urinary DAP measure is less representative of longer-term exposure than are serial measurements. In addition, DAP metabolites in urine may in part reflect exposure to preformed DAPs present in the environment or food (Lu et al. 2005); therefore, the proportion of urinary DAP metabolites that reflect exposure to parent pesticide compounds is unknown. However, these sources of exposure misclassification are nondifferential and would bias results toward the null. Despite the limitations pertaining to the use of urinary DAPs as exposure indicators to OP pesticides, they may provide the best integrated measure available at this time. Indeed, for many OP pesticides, no methods currently available measure pesticide-specific metabolites in urine or OP parent compounds in blood.

Prenatal exposure to OP pesticides, primarily with DM rather than DE metabolites, was associated with poorer cognition at 2 years of age in this cohort (Eskenazi et al. 2007), as well as in the 7-year follow-up we report here. The exception was that DE metabolites were more strongly associated than DM metabolites with deficits on Processing Speed. The stronger associations with DM metabolites for most cognitive measures could be explained by the greater toxicity of some of these OP pesticides. For example, oxydemeton-methyl, which devolves to DM metabolites, is the most toxic of OP pesticides used in the study region and represents the greatest cumulative risk (Castorina et al. 2003). On the other hand, DE metabolites may be less stable and, consequently, poorer exposure biomarkers (Bradman et al. 2007); this would likely bias the effect estimates toward the null.

The present study also has considerable strengths, perhaps most notable among them being its longitudinal design. We measured urinary DAP concentrations during prenatal development and throughout childhood. We followed children until 7 years of age, when the tests of cognitive function are more reliable than at younger ages (Honzik 1976). As in any epidemiologic study, the reported associations could be attributable to uncontrolled confounders, but we were able to examine or adjust for numerous important factors over the lifetime of the child, including exposure to other environmental agents, several socioeconomic indicators, maternal cognitive abilities, and child stimulation. Urinary DAP concentrations during pregnancy were weakly associated with measures of socioeconomic status, such as maternal intelligence and education. Furthermore, the study population had a homogeneous socioeconomic profile, reducing the potential for uncontrolled confounding.

The level of urinary DAP metabolites in the pregnant women in the present study was higher than in a representative U.S. sample of women of reproductive age [National Health and Nutrition Examination Survey (NHANES) 1999–2000] (Bradman et al. 2005). In the present group, the median of total maternal DAP concentrations was 128 nmol/L. As a comparison, NHANES levels were 72 nmol/L among pregnant women and 90 nmol/L among nonpregnant women. However, > 25% of pregnant women from the general U.S. population had DAP levels exceeding the median levels measured in the present study. Thus, the prenatal DAP concentrations associated with cognitive deficits in offspring in the present investigation were within the range of concentrations found in the general population.

## Conclusion

Prenatal but not postnatal exposure to OP pesticides was associated with poorer intellectual development in 7-year-old children from an agricultural community. Maternal urinary DAP levels in this sample, although higher than general U.S. levels, were nonetheless within the range of the distribution levels. These findings suggest that some U.S. women in the general population may experience OP pesticide exposure at levels that are associated with poorer cognitive development in offspring in the present study.

## Correction

In the original manuscript published online, some of the values in Table 4 were incorrect. They have been corrected here.

## Supplemental Material

(48 KB) PDF
