# Supplementary material for: Prenatal Exposure to Organophosphate Pesticides and IQ in 7-Year-Old Children
Source: Environ Health Perspect. 2011 Apr 21;119(8):1189–95. doi: 10.1289/ehp.1003185 (PMC3237357; doi:10.1289/ehp.1003185)
Supplement: (48 KB) PDF [file ehp.1003185.s001.pdf]

Supplemental Material

**Prenatal Exposure to Organophosphate Pesticides and IQ in 7-Year Old Children**

Maryse F. Bouchard, Jonathan Chevrier, Kim G. Harley, Katherine Kogut, Michelle Vedar, Norma Calderon, Celina Trujillo, Caroline Johnson, Asa Bradman, Dana Boyd Barr, Brenda Eskenazi

Supplemental Material, Table 1: Change in cognitive scores for a 10-fold increase in prenatal creatinine-adjusted DAP, DM, and DE concentrations, CHAMACOS

| Cognitive scores | | DAP | | DM | | DE | |
|---|---|---|---|---|---|---|---|
| | N | β (95% CI) | p | β (95% CI) | p | β (95% CI) | p |
| WISC-IV scales | | | | | | | |
| Working Memory | 329 | -3.8 (-7.1, -0.5) | .03 | -3.5 (-6.5, -0.6) | .02 | -0.1 (-3.1, 3.0) | .97 |
| Processing Speed | 329 | -3.6 (-6.9, -0.4) | .03 | -2.0 (-4.9, 0.9) | .19 | -4.4 (-7.3, -1.5) | .01 |
| Verbal Comprehension | 298 | -4.8 (-8.0, -1.6) | .01 | -4.3 (-7.2, -1.4) | .01 | -2.1 (-5.1, 0.9) | .17 |
| Perceptual Reasoning | 298 | -4.8 (-8.3, -0.7) | .02 | -3.6 (-7.0, -0.2) | .04 | -2.9 (-6.4, 0.6) | .10 |
| Full Scale IQ | 297 | -5.3 (-8.6, -2.0) | .01 | -4.3 (-7.3, -1.3) | .01 | -3.1 (-6.1, -0.0) | .05 |

Estimates were adjusted for HOME at 6 months, maternal education and intelligence. Verbal Comprehension and Full Scale IQ were also adjusted for language of assessment.

Supplemental Material, Table 2: Change in cognitive scores for a 10-fold increase in prenatal creatinine-adjusted DAP concentrations, restricting the analytical sample to children assessed in Spanish, CHAMACOS

| Cognitive scores | | DAP | |
|---|---|---|---|
| | N | β (95% CI) | p |
| WISC-IV scales | | | |
| Working Memory | 221 | -4.4 (-8.5, -0.3) | .04 |
| Processing Speed | 221 | -4.3 (-8.3, -0.3) | .04 |
| Verbal Comprehension | 198 | -5.3 (-9.7, -0.9) | .02 |
| Perceptual Reasoning | 198 | -5.6 (-10.4, -0.7) | .02 |
| Full Scale IQ | 197 | -6.2 (-10.5, -1.8) | .01 |

Estimates were adjusted for HOME at 6 months, maternal education and intelligence.

Supplemental Material, Table 3: Change in cognitive scores for a 10-fold increase in DAP concentrations (nmol/L) measured prenatally and postnatally, CHAMACOS

| Cognitive scores | | Prenatal DAP | | Postnatal DAP | | p-value for slopes comparison |
|---|---|---|---|---|---|---|
| | N | β | (95% CI) | β | (95% CI) | |
| WISC-IV scales | | | | | | |
| Working Memory | 278 | -3.7 | (-7.3, -0.1) | 0.5 | (-3.2, 4.2) | .10 |
| Processing Speed | 278 | -4.4 | (-7.9, -0.9) | -1.4 | (-5.0, 2.3) | .24 |
| Verbal Comprehension | 308 | -5.4 | (-9.0, -1.9) | 1.3 | (-2.4, 4.9) | .01 |
| Perceptual Reasoning | 308 | -3.5 | (-7.7, 0.6) | 0.2 | (-4.1, 4.5) | .19 |
| Full Scale IQ | 277 | -5.5 | (-9.2, -1.8) | 0.0 | (-3.8, 3.9) | .03 |

Estimates were adjusted for HOME score at 6 months, maternal education and intelligence. Verbal Comprehension and Full Scale IQ were also adjusted for language of assessment.
